# Knowledge and perception of labor rights violations among postpartum women in three Brazilian maternity hospitals: a cross-sectional study

**DOI:** 10.1186/s12884-025-08317-1

**Published:** 2025-11-14

**Authors:** Aline Munezero, Julia M. Fein, Marta P. Nhauche, Karayna G. Fernandes, Melissa Y. Takayama, Débora FB Leite, Guilherme Figueiredo, Renato Passini, Maria Jose MD Osis, Jose G. Cecatti, Renato T. Souza

**Affiliations:** 1 Department of Obstetrics and Gynecology, University of Campinas, 101 Alexander Fleming st, Cidade Universitaria, Campinas, SP, ZIPCODE 13083-881 Brazil; 2 Jundiaí School of Medicine - HU/FMJ, 250 Francisco Teles st, Vila Arens, Jundiaí, SP, ZIPCODE 132202-550 Brazil; 3 Federal University of Pernambuco - HC/UFPE, 1235 Prof. Moraes Rego avenue, Cidade Universitaria, Recife, PE, ZIPCODE 50670-901 Brazil

**Keywords:** Labor rights, Violation, Pregnancy, Women’s right

## Abstract

**Background:**

The study explores the knowledge and perception of labor rights violations among the obstetric population in Brazil, highlighting the significant role of laws and policies in women’s reproductive health.

**Methods:**

A cross-sectional multicenter study in three Brazilian maternity facilities in June-July 2022. Postpartum women were included and responded to a questionnaire about their knowledge of labor rights; those who worked during pregnancy answered about their perception of violations of labor rights. Multivariate analysis was used to identify factors associated with level of knowledge and perception of violation of LR.

**Results:**

652 postpartum women were enrolled and 293 who worked during pregnancy answered about rights violations; 8.1% women knew all their labor rights, and 40.8% did not know at least half of the 16 specified labor rights. Being adolescent (ORadj: 2.12 [1.22–3.69] 95% CI), living in the Brazilian Northeast region (ORadj: 3.41 [2.36–4.92] 95% CI), having low education (ORadj: 1.88 [1.26–2.81] 95% CI) and being a single mother (ORadj: 1.80 [1.24–2.61] 95% CI) were associated with limited knowledge of labor rights. Over 50% of pregnant women had their labor rights violated. Women from the northeast region of Brazil were five-fold more likely to experience rights violation (ORadj: 5.35 [2.52–11.52] 95% CI).

**Conclusion:**

Few pregnant women knew their rights, and many experienced violations of labor rights, especially socially vulnerable women. Better health education on labor rights and increased monitoring are needed to protect women´s rights.

**Supplementary Information:**

The online version contains supplementary material available at 10.1186/s12884-025-08317-1.

## Background

The most updated concept of maternal and perinatal health is shaped by the multidimensional view that included not only essential care so that the pregnancy does not present complications, but also consider guaranteeing the pregnant woman a positive experience [1]. Laws and policies are critical layers in the women’s reproductive health cycle, which can have a significant influence for complications and the chance to get full and healthy recovery from pregnancy and postpartum periods [2]. The guarantee of this experience is related to fundamental rights and policies that ensure collective protection to women’s rights reducing gender inequities [1]. However, the recognition of women’s rights occurred slowly and progressively Wollstonecraft [3]. In 1975, the UN convened a World Conference on Women, and in this event, a legal instrument was created that safeguarded women’s rights in the world. And consequently, in 1979, there was the creation of CEDAW - “Convention on the Elimination of All Forms of Discrimination Against Women” [4].

Given the importance of work in women’s lives, labor rights are essential to guarantee workers’ well-being - which includes several specificities for women, such as in the postpartum pregnancy period [5]. The pregnancy and postpartum period is considered a unique period, and a woman’s (and their partner’s lives) memorable experiences, as well as for their relatives [6, 7]. Moreover, during this period, the woman undergoes several psychological and physiologic changes, which demand woman adaptation to these new events, especially for women who work. Therefore, women must be protected by laws that ensure their dignity and respect in the labor market [6, 7].

Labor rights are country-specific. In Brazil, labor rights are reported in the 1943 Consolidation of Labor Laws (CLT). The CLT “establishes the norms that regulate individual and collective labor relations, between the employer and employee” [8, 9]. Articles currently directed at all workers, such as the 5th, establish equal pay for the same work, regardless of gender. Remarkably, there is a particular chapter for “Protection of women’s work” (Chapter III), aimed exclusively at female workers, in addition to section V “, maternity protection”, which ensures the rights of pregnant women in the labor market [8, 9].

However, violation or lacking labor rights can lead to several negative impacts [10, 11]. An example of this is part-time contracts, which are more accepted by women (due to lack of option or imposition of the employer), accounting for 41.2% of women’s jobs [10, 11]. This type of contract, along with temporary contracts and outsourcing, present gender bias, stimulating the flexibilization of female contracts, since they are forms of employment that generally generate less social protection, denying certain rights – which generates strong impacts at the time of pregnancy, because the provisional stability of pregnant women is not protected, and these contracts are preferred by employers who still discriminate against women [10, 11].

Women’s knowledge about their labor rights, especially during pregnancy, can ensure adequate antenatal care provision, including a sufficient number of consultations and a safer work environment, ensuring change of workplace or activity if it poses a risk to the pregnancy [12]. Studies have suggested that pregnant women have limited knowledge of fundamental women’s labor rights, such as paid maternity leave for adoptive mothers and mothers with preterm babies [12]. However, data on the degree of knowledge and the burden of labor rights violations among pregnant and postpartum women are scarce. Evidence regarding the labor rights violations on maternal and perinatal health remains unclear. Given this unmet need, the present study aimed to measure the degree of knowledge and perceived violation of maternal labor rights in three Brazilian maternity hospitals.

## Methods

We conducted a multicenter cross-sectional study from June 1 to July 31 2022 including postpartum women from three Brazilian maternity hospitals - Hospital da Mulher Prof. Dr José Aristodemo Pinotti - CAISM, Campinas, SP; University Hospital of the Faculty of Medicine of Jundiaí, Jundiaí, SP, Clinics Hospital of the Federal University of Pernambuco, Recife, PE. Daily surveillance (or, at least, on alternate days) by the research team was implemented at recruitment sites to identify eligible women. At the time of inclusion, post-partum women were assessed for eligibility according to the following criteria. Inclusion criteria: women hospitalized due to delivery or postpartum care at the participating centre. Exclusion criteria were having had a stillbirth in the current pregnancy or a history of retirement due to disability. Participants were included in the study after being informed about the research and having signed the consent form. Underaged women were also considered eligible and they invited to participate and, in these cases, the legal guardian signed the consent form.

### Sample size calculation

By the time of the study implementation, there were no studies evaluating the degree of knowledge of labor rights in the pregnant population; therefore, our sample size calculation was based on finding from the MAES study (resilience during pregnancy) [13], and the final sample size was 580 participants. However, the post-hoc power analysis based on the study population indicated a sample power of 99.9% and 72.8% for the assessment of knowledge of labor rights, considering the estimated proportion of the categorical variable (degree of knowledge), the type I error at 5% (alpha = 0.05) or 95% confidence interval, the sampling error at 5% (d = 0.05) and the sample size (*n* = 652).

### Data collection

We used an instrument to assess knowledge about labor rights and the degree of perceived violation of labor rights reported by postpartum women. The questionnaire about labor rights was comprised of some general rights for Brazilian workers and specific rights for pregnant women (Supplementary Material; Table S1). In Brazil, these laws are described in the CLT (Consolidation of Labor Laws) [14]. The CLT has more than 900 articles divided into nine titles (Title I - Introduction (Articles 1 to 12); Title II - General Norms for Labor Protection (Articles 13 to 223); Title III - Special Norms for Labor Guardianship (articles 224 to 441); Title IV – Rural Work (articles 442 to 510); Title V – Union Organization (articles 511 to 610); Title VI – Collective Bargaining Agreements (articles 611 to 625); Title VII - The Process of Labor Administrative Fines (Articles 626 to 642); Title VIII - The Labor Court (Articles 643 to 735); Title IX - The Public Ministry of Labor (Articles 736 to 762); Title X - The Judicial Process of Labor (Articles 763 to 910); Title XI – Final and Transitional Provisions (Articles 911 to 922). In addition, section V (Protection for Maternity) of Chapter III (Protection of Women’s Work) of Title III (Special Guardianship Regulations) of labor has 15 articles, including several labor rights in its items and paragraphs.

The list of labor rights was elaborated by an expert committee composed of professors and senior researchers (3 obstetricians and gynecologists, a lawyer, and a sociologist). Specialists (two OB-GYNs, a legal scholar, and social science and anthropology scientist) selected the labor right. After discussion, 16 labor rights were selected (ten labor rights among five articles of the Maternity Protection section and six rights related to 6 articles considered “General Rights”). During the interview, women reported which rights they were aware of (they knew the right) and which rights were violated. The evaluated the degree of violation of rights against women working under a CLT regimen during pregnancy. Women reported which rights were violated. We considered only women who worked during pregnancy to assess the labor rights violation. In addition, we evaluated only evaluated violation of 15 out of 16 labor rights (Article 395 was removed as it was related to abortion). We considered all 15 labor rights for multiparous but only nine labor rights for nulliparous (Supplementary Material; Table S1). We did not assess the violation of labor rights regarding maternity leave and breastfeeding for nulliparous.

We performed face-to-face interviews in the postpartum period, during the woman’s hospitalization (after delivery and before hospital discharge). Data collection instruments were self-completed and/or applied by the interviewer. These data collection instruments were developed in the REDCap^®^ platform, an online database system with offline access through tablets or mobile phones, with restricted and hierarchical access, greater privacy and confidentiality of the data. In addition, this software allows dynamic data management, increasing research quality, ensuring accuracy, security and speed [15].

### Data management and statistical analysis

Categorical variables were presented as a measure of prevalence, and the χ2 test or Fisher’s exact test were used for comparison. Moreover, the degree of knowledge and perceived violation were transformed into dichotomous categorical variables (yes/no). The variables of measurement of the degree of knowledge and perception of violation were converted into scores from 0 to 100%, according to the percentage of rights surely known by the participants and those reported as being violated. In total, we assessed knowledge of sixteen rights, and violation of fifteen rights for multipara and nine rights for nullipara. Only women who worked during pregnancy were considered.

Considering that we do not know exactly the proportion of women that know their rights and what would be an acceptable cut-off, to analyze the association between maternal characteristics and level of knowledge of labor rights, we compared categories based on population´s distribution based on the quartiles (degree of knowledge greater than the Q3, between Q1 and Q3 (inclusive) and below the Q1). Likewise, we also compared those who knew more or less than 50% of the selected rights (knowledge greater than or equal to 50% of the rights and below 50% of the labor rights).

The perception of violation was categorized by the proportion of rights violated (at least one versus none; at least one-third versus less than one-third of labor rights violated).

Factors independently associated with a low degree of knowledge of labor rights (< 50%) and the perception of rights violation (at least one right violated) were identified using multiple regression analysis (Backward stepwise regression model). The following predictors were considered: region [Northeast = 1, Southeast = 0]; ethnicity [black = 1, other = 1, white = 0]; age [< 19 = 1, 20–35 = 1, > 35 = 0], schooling [elementary/middle = 1, high school = 0, university = 0], family monthly income [< 1 = 1, 1–3 = 1, > 3 = 0], having a partner [having a partner = 0, not having a partner = 1]. For the predictive model of the perception of rights violated, we also considered the degree of knowledge of labor rights. We estimated the Crude and adjusted Odds Ratio (ORadj), and its confidence intervals and *p*-values.

Statistical analyses were performed using IBM SPSS^®^ software. Therefore, we considered p-<0.05 for the level of statistical significance.

### Ethical aspects

The study received approval from all the Institutional Review Boards (IRB) from each participating centers (IRBs: School of Medical Sciences, University of Campinas in Campinas; Jundiaí Medical School in Jundiai; Center for Health Sciences of the Federal University of Pernambuco in Recife). The approval letters were issued on August 03, 2021. Eligible women were invited to participate in the study and provided their consent by signing an informed consent form. The study adhered to the methodological procedures and ethical considerations outlined in the Declaration of Helsinki (amended in Hong Kong in 1989) and the ethical principles of the Brazilian National Health Council (Resolution CNS 466/12). The study participants were de-identified, and data was collected and stored on the REDCap platform to ensure anonymity. All women assessed for eligibility received a flyer containing information on labor rights; the document contained a description of selected rights and a qr-code linked with the official website of the Brazilian national labor rights legislation.

## Results

**Fig. 1:**
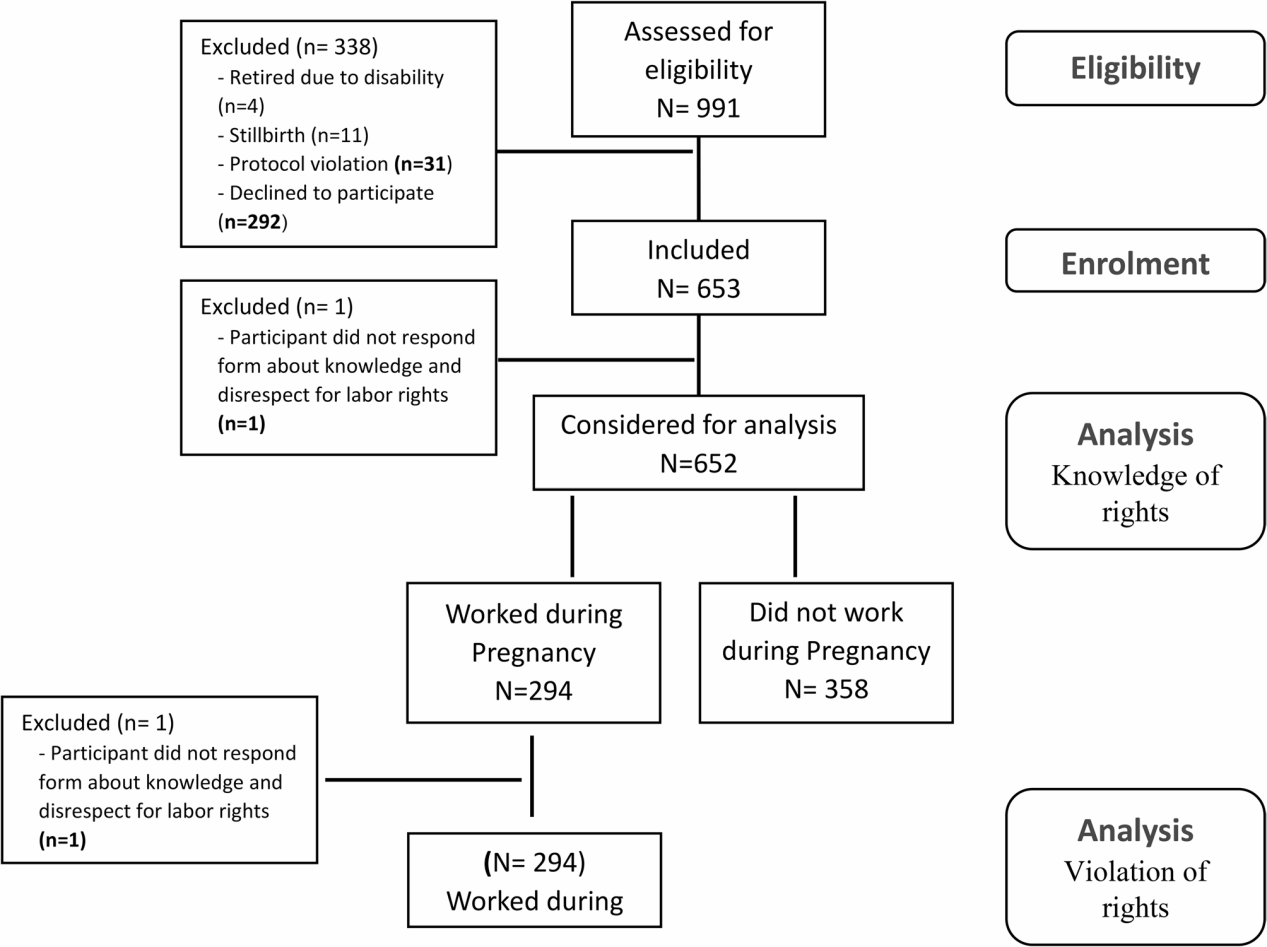
Flowchart of the participants considered for analysis

We screened 991 postpartum women for eligibility, 652 participants were included; and 338 (34.1%) women did not accept the invitation to participate (Fig. 1). From the 294 women who worked during pregnancy, 293 answered about their perception of violation of labor rights.

Out of the 652 postpartum women interviewed, 458 (70.2%) were from the Southeast and 194 (29.7%) from the Northeast. The age of the participants ranged from 12 to 47 years, with a mean of 27.8 years (SD = ± 7 years). In addition, 445 (68.2%) were black, 246 (37.7%) had a partner, 383 (58.7%) had high school education, 113 (17.3%) had higher education, and 294 (45%) worked during pregnancy. About 135 (20.7%) and 342 (52.4%) of the participants reported family income of up to 1 and 1–3 minimum wages, respectively. From those who worked during pregnancy (*n* = 294), 115 (39.1%) worked informally, and 142 (48.3%) worked more than 40 h per week. (Table 1).

**Table 1:** Degree of knowledge about labor rights related to characteristics of postpartum women (*N* = 652)

Characteristics	Limited knowledge about labor rights (< Q1) *N* (%)	Greater knowledge about labor rights (≥ Q1) *N* (%)	*p*-value*
Region			< 0.001
Southeast	63 (40.7)	395 (75.5)	
Northeast	92 (59.4)	102 (20.5)	
Age			< 0.001
< 19	37 (23.9)	44 (8.9)	
20–35	99 (63.9)	360 (72.4)	
> 35	19 (12.3%)	93 (18.7)	
Ethnicity			< 0.001
Black	125 (80.6)	310 (62.4)	
White	28 (18.1)	179 (36.0)	
Other	2 (1.3)	8 (1.6)	
Marital status			< 0.001
Without a partner	121 (78.1)	285 (57.3)	
With a partner	34 (21.9)	212 (42.7)	
Schooling			< 0.001
Elementary/Middle	60 (38.7)	96 (19.3)	
High school	89 (57.4)	294 (59.2)	
University	6 (3.9)	107 (21.5)	
Family Monthly income*			< 0.001
Below 1	59 (39.6)	76 (15.9)	
1–3	78 (52.3)	264 (55.3)	
*≥* 3	12 (8.1)	137 (28.7)	
Worked during pregnancy			< 0.001
No	121 (78.1)	237 (47.7)	
Yes	34 (21.9)	260 (52.3)	
Total	155	497	

**Fig. 2:**
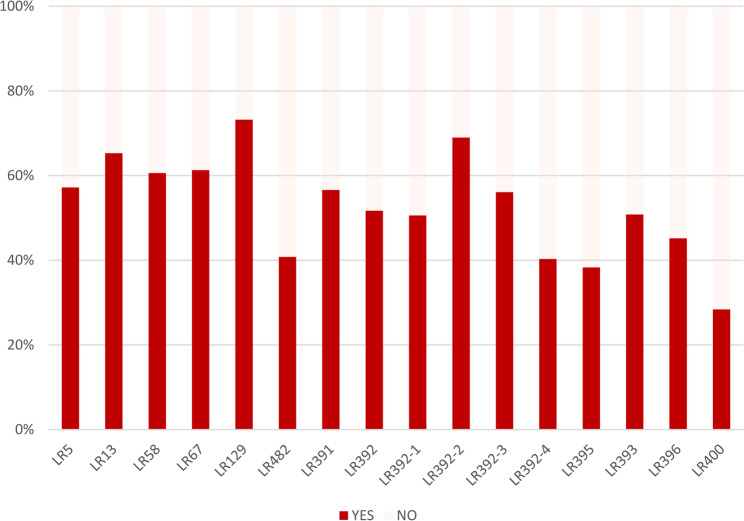
Knowledge about sixteen selected labor rights (*n* = 652)

Figure 2 shows the proportion of women who reported that they knew each of the sixteen selected rights. The LR129 was the most widely known right by study participants (73.2%) [*Art. 129. Every employee will be annually entitled to a vacation*,* without the loss of remuneration*], followed by the LR392-2 (69%) [*Art. 392. The pregnant employee is entitled to take maternity leave of 120 (one hundred and twenty) days*,* without termination of employment or salary*]. Conversely, the LR400 was the least known right (28.4%) [*Art. 400. Places that provide childcare for children of female employees*,* during the breastfeeding period*,* must have at least one nursery*,* a breastfeeding room*,* a dietetic kitchen and a sanitary facility*] (Fig. 2).

**Fig. 3:**
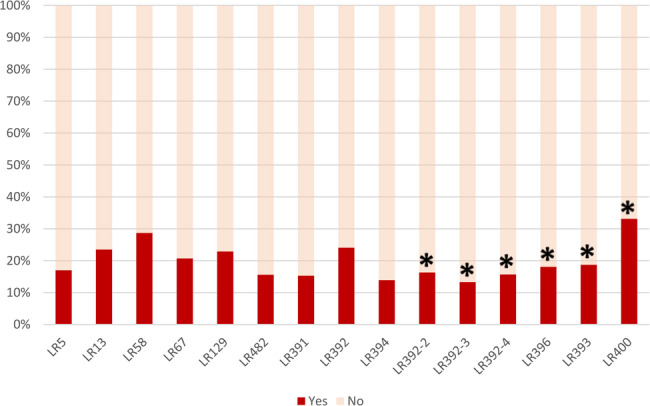
Perception of violation for fifteen labor rights (*n* = 293)

**Fig. 4:**
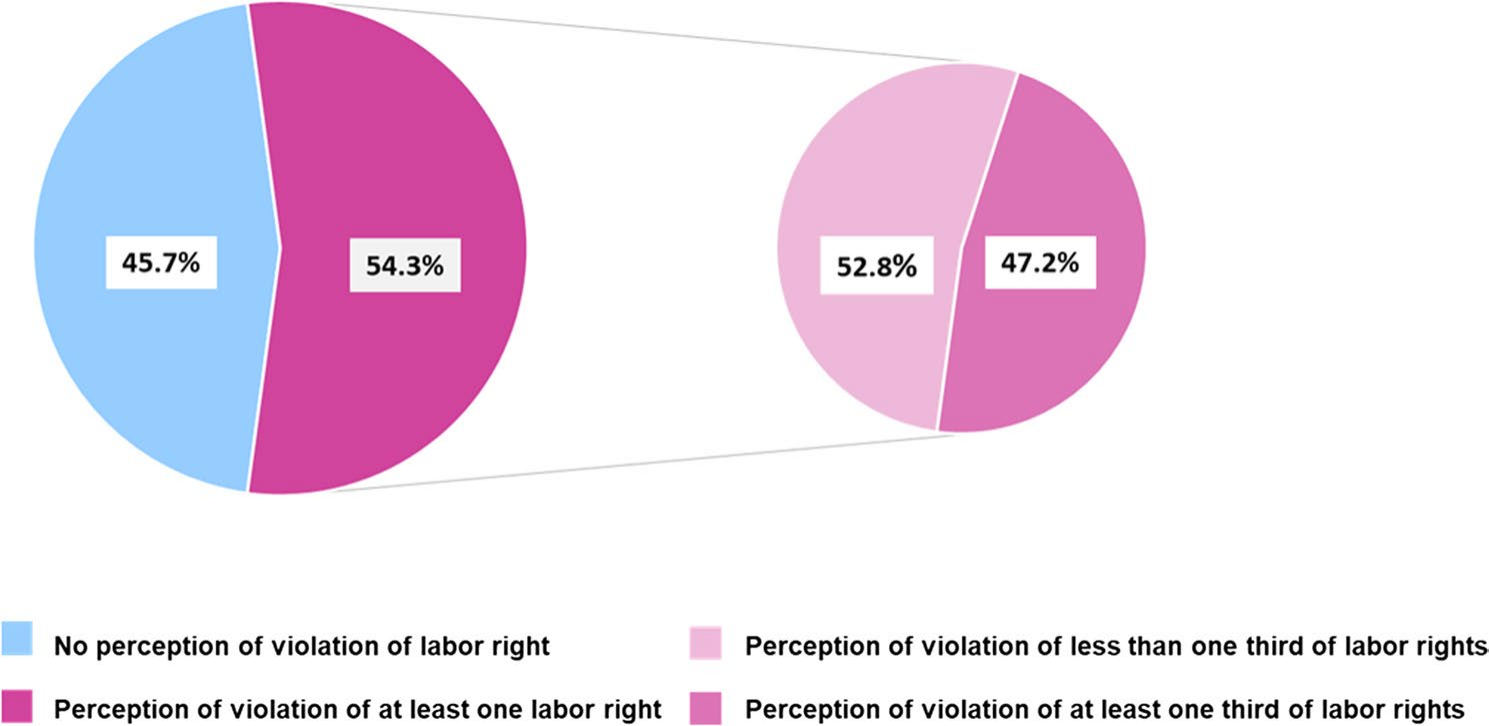
Proportion of women reporting violation for fifteen selected labor rights (*N* = 293). * Statistically significant (*p*-value < 0.05)

Figure 3 shows the proportion of perception of violation of right for each of the selected rights. It ranged from 13% (LR392-3) to 33% (LR400). Regarding the perception of violation of labor rights, 54,3% (159/294) of the participants reported violation of at least one labor right (Fig. 4); from those women, 47,2% (75/159) reported violation of at least one-third of the selected rights (Fig. 4).

Univariate analysis showed a significant association between the degree of knowledge of labor rights and different sociodemographic characteristics (Tables 1 and 2). Also, we found that education, region and degrees of knowledge of labor rights were significantly associated with the violation of at least one third of labor rights in the univariate analysis (*p*-value < 0.001) (Table 3). Similarly, region (*p*-value < 0.001) and degree of knowledge about labor rights (*p*-value = 0.013) were factors significantly associated with the perception of violation of at least one labor right (Table 4).

**Table 2:** Degree of knowledge about labor rights related to characteristics of postpartum women (*N* = 652)

Characteristics	Know less than 50% of the selected rights *N* (%)	Know more than 50% of the selected rights *N* (%)	*p*-value
Region			< 0.001
Southeast	145 (54.5)	313 (81.1)	
Northeast	121 (45.5)	73 (18.9)	
Age			< 0.001
< 19	51 (19.2)	30 (7.8)	
20–35	182 (68.4)	277 (71.8)	
> 35	33(12.4)	79 (20.5)	
Ethnicity			< 0.001
Black	190 (71.4)	245 (63.5)	
White	72 (27.1)	135 (35.0)	
Other	4 (1.5)	6 (1.6)	
Marital status			< 0.001
Without partner	195 (73.3)	211 (54.7)	
With partner	71 (26.7)	175 (45.3)	
Schooling			< 0.001
Elementary/Middle	84 (31.6)	72 (18.7)	
High school	160 (60.2)	223 (57.8)	
University	22 (8.3)	91 (23.6)	
Minimum income wages ^a^			< 0.001
< 1	81 (31.8)	54 (14.6)	
1–3	134 (52.5)	208 (56.1)	
> 3	40 (15.7)	109 (29.4)	
Worked during pregnancy	82 (30.8)	212 (54.9)	< 0.001
Total	266	386

**Table 3:** Perception of labor rights violation by women who worked throughout pregnancy in relation to characteristics of postpartum women (*N* = 293)

Characteristics	At least one third of rights (*N* %)	Less than one third or None (*N* %)	*p* value*
Region			< 0.001
Southeast	50 (66.7)	188 (86.2)	
Northeast	25 (33.3)	30 (13.8)	
Age			0.812
< 19 years old	7 (9.3)	17 (7.8)	
20–35 years	56 (74.7)	160 (73.4)	
> 35 years old	12 (16.0)	41 (18.8)	
Ethnicity			0.063
Black	55 (73.3)	129 (59.2)	
White	18 (24.0)	85 (39.0)	
Other	2 (2.7)	4 (1.8)	
Marital status			0.834
Without partner	43 (57.3)	128 (58.7)	
With partner	32 (42.7)	90 (41.3)	
Schooling			< 0.001
Elementary/Middle	20 (26.7)	31 (14.2)	
High school	47 (62.7)	122 (56.0)	
University	8 (10.7)	65 (29.8)	
Minimum income wages			0.019
Until 1	12 (16.2)	14 (6.7)	
1–3	43 (58.1)	115 (55.0)	
> 3	19 (25.7)	80 (38.3)	
Knowledge about labor rights			< 0.001
< Q1 (< 32.1%)	20 (26.7)	14 (6.4)	
Q1-Q3 (32.1–81.2%)	40 (53.3)	136 (62.8)	
>Q3 (81.2%)	15 (20.0)	67 (30.7)	
Total	75	218

**Table 4:** Perception of labor rights violation by women who worked throughout pregnancy in relation to characteristics of postpartum women (*N* = 293)

Characteristics	At least one labor right *N* (%)	None (*N* %)	*P*-value
Region			< 0.001
Southeast	113 (71.1)	125 (93.3)	
Northeast	46 (28.9)	9 (6.7)	
Age (years)			0.687
< 19	11 (6.9)	13 (9.7)	
20–35	119 (74.8)	97 (72.4)	
> 35	29 (18.2)	24 (17.9)	
Ethnicity			0.490^*^
Black	109 (68.5)	75 (55.9)	
White	46 (28.9)	57 (42.5)	
other	4 (2.5)	2 (1.5)	
Marital status			0.106
Without a partner	86 (54.1)	86 (63.4)	
With a partner	73 (45.9)	49 (36.6)	
Schooling			0.254
Elementary/Middle	31 (19.5)	20 (14.9)	
High school	94 (59.1)	75 (55.9)	
University	34 (21.3)	39 (29.1)	
Minimum income wages ^a^			0.142
Until 1	19 (12.1)	7 (6.0)	
1–3	87 (55.4)	71 (56.3)	
> 3	51 (32.5)	48 (38.1)	
Knowledge about labor rights			< 0.013
< Q1 (< 32.1%)	25 (15.7)	9 (6.7)	
Q1-Q3 (32.1–81.2%)	98 (61.6)	79 (58.9)	
>Q3 (81.2%)	36 (22.6)	46 (34.3)	
Total	159	134	

*Fisher’s Exact Test; Missing information for a) 10

**Fig. 5:**
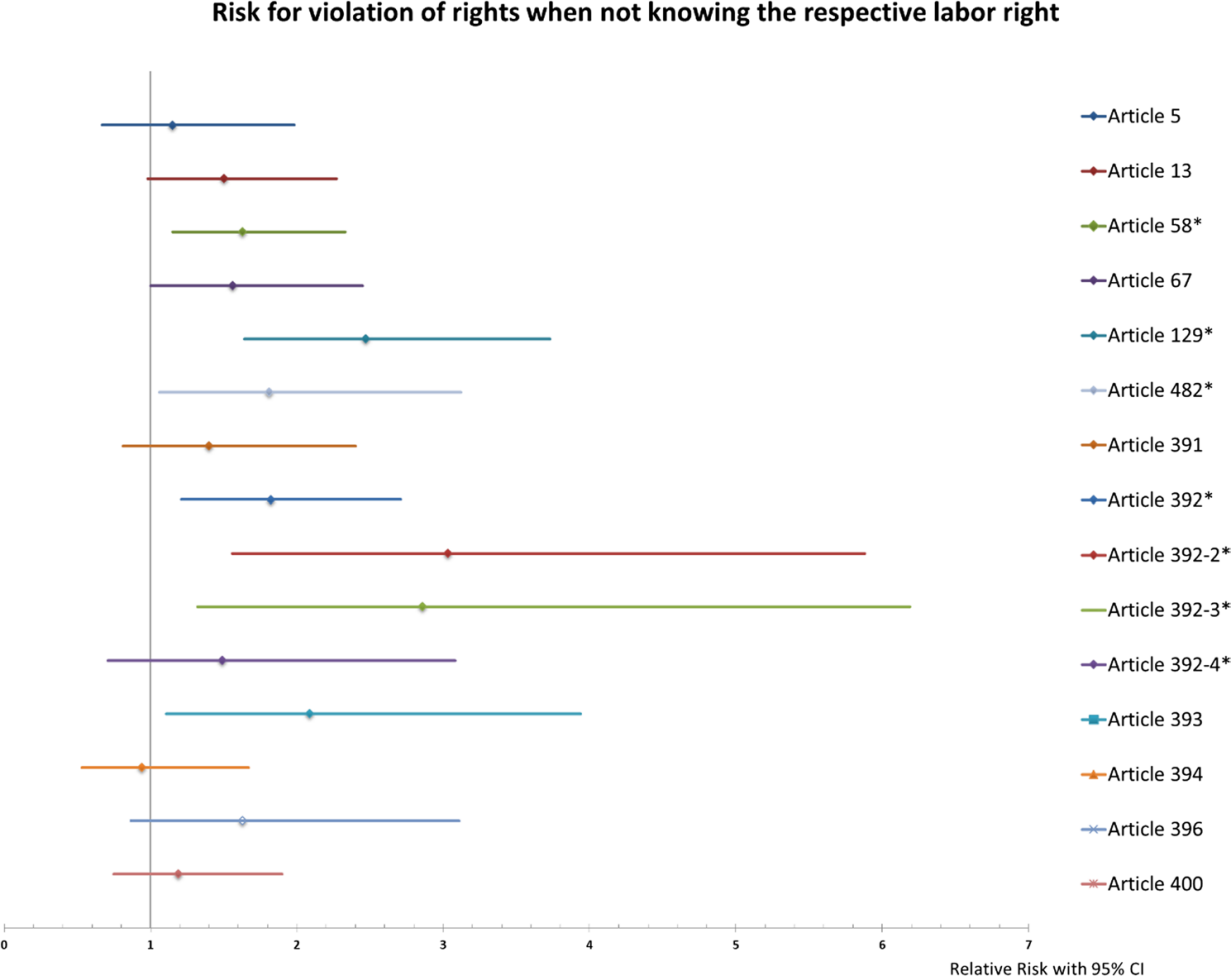
Risk of perceived violation for labor rights according to knowledge about the respective right

We assessed the risk for violation of labor right according to the status of knowledge of the respective labor right. Participants that reported limited knowledge about LR58, LR129, LR392, LR392-2, and LR392-3, were more likely to perceive disrespect of these labor rights (Fig. 5).

According to the multiple regression analysis, not having a partner (OR_adj_: 1.80, 95% CI [1.24–2.61]), low educational level (OR_adj_: 1.88, 95% CI [1.26–2.81]), being adolescent (OR_adj_: 2.12 95% CI [1.22–3.69]), and living in the Northeast region (OR_adj_: 3.41, 95% CI [2.36–4.92]) were factors independently associated with limited knowledge about labor rights (< 50% of the selected rights) (Table 5). Living in the Northeast region was also independently associated with perceived violation of at least one labor right (OR_adj_: 5.38, 95% CI [2.52–11.52]) (Table 6).

**Table 5:** Factors independently associated with limited knowledge about employment rights (< 50% of the selected rights) (*n* = 626)

Variables	ORadj	95% CI	*p*-value
Not having a partner	1.80	1.24–2.61	0.002
Schooling (Elementary school)	1.88	1.26–2.81	0.002
Age (< 19y)	2.12	1.22–3.69	0.007
Region (Northeast)	3.41	2.36–4.92	< 0.001

Initial predictors entering the model: region (northeast = 1, southeast = 0); ethnicity (black = 1, other = 1, white = 0); age (< 19 = 1, 20–35 = 0, > 35 = 0), schooling (elementary/middle = 1, high school = 0, university = 0), family monthly income (≤ 1 wage = 1, > 1 wage = 0), having a partner (Having a partner = 0, not having a partner = 1)

**Table 6:** Factors independently associated with the perception of violation for at least one labor right (*n* = 283)

Variables	ORadj	95% CI	*p*-value
Region (Northeast)	5.38	2.52–11.52	< 0.001

Initial predictors entering the model: region (northeast = 1, southeast = 0); ethnicity (black = 1, other = 1, white = 0); age (< 19 = 1, 20–35 = 0, > 35 = 0), schooling (elementary/middle = 1, high school = 0, university = 0), family monthly income (≤ 1 wage = 1, > 1 wage = 0), having a partner (Having a partner = 0, not having a partner = 1), knowledge about selected labor rights (< 50%=1, ≥ 50%=0)

## Discussion

Our findings have suggested that when considering labor rights, women have more knowledge about labor rights (such as Art. 129, which states that “Every employee shall be annually entitled to a vacation, without the loss of remuneration” and 392 stating that “Pregnant workers are entitled to maternity leave of 120 (one hundred and twenty) days, without the loss of employment or salary” [8]. Furthermore, sociodemographic factors (not having a partner, low education level, being adolescent and living in the Northeast region had a great influence on the degree of knowledge of labor rights. Proper implementation of these laws allows postpartum women a reasonable time to adapt to physiological changes inherent to the postpartum period and greater availability to offer adequate neonatal care during maternity and annual leave without losing their salaries, thus reducing the socioeconomic vulnerability of this group.

However, regarding labor laws in the pregnancy-puerperal cycle, our study showed that law 16 is less well-known (“*Art. 400 Places that provide childcare for employees’ children*,* during the breastfeeding period*,* should have at least one nursery*,* a breastfeeding room*,* a dietetic kitchen and a sanitary facility”)* [8]. Common knowledge of this right or lack of this benefit can lead mothers, children, and society in general to miss out on the health benefits of breastfeeding.^3,22^ When Brazilian women who are employed in the formal labor market return to work after childbirth, they are entitled to take two half-hour breaks during the working day to breastfeed their babies until he/she is six months old. Having more knowledge about these employment rights and implementation of this important public health policy are associated with lower morbidity and longer child survival, especially in socially vulnerable family groups with a lower income.

Compliance with breastfeeding protection laws is fundamental in a society similar to ours in Brazil, where almost 25% of the economically active population consists of women with children under six months of age and 92% of women who are not offered the benefit of workplace daycare introduce breast milk substitutes [14, 16].

The right to breastfeed after returning to work is essential for maternity protection and mother-baby bonding. Nevertheless, companies need to comply with the law so that women can enjoy their rights. Therefore, it is necessary to supervise and penalize employers that fail to comply with labor laws.

On multiple regression analysis, variables related to a low degree of knowledge about labor rights were determining factors for a higher risk of social vulnerability. The factors identified were low educational level, no partner, and living in the Northeast region of Brazil, where the human development index (HDI) is the lowest in the country. These findings suggest that socially disadvantaged women with a low level of education are at a higher risk of suffering violation of labor rights, a high chance of social injustice and an increased risk of adverse outcomes during the pregnancy-puerperal cycle [17, 18].

The low educational level of women (less than five years of schooling), considered by the Minister of Health as a risk factor for pregnancy, can also hinder the development of educational activities promoted by health services due to difficulty in understanding [19, 20]. Women with a university degree have a 35% drop in employment after 12 months of leave, while women with lower levels of schooling have a 51% decrease [19, 20]. Thus, our study shows the importance of providing information on legally established labor rights to all employees, especially pregnant and postpartum women, regardless of their educational level. Women with adequate and timely access to information about labor laws can better monitor and demand full implementation of their rights, as well as require that corporations duly comply with labor benefits.

Findings from a previous study has suggested that women who know their labor rights during pregnancy can guarantee access to prenatal care, request a change of place or activity that may jeopardize the pregnancy and exclusively breastfeed their baby for a longer time. These measures prevent health complications during pregnancy and the postpartum period [19].

Regarding the perception of pregnancy discrimination in the workplace, more than half of the participants reported that at least one labor right was violated. Despite the existence of labor laws, discrimination against women during pregnancy and the puerperium is still common. Unfortunately, it is thought that hiring women can generate higher costs and less dedication. According to a survey of 247,000 women, 24 and 47 months after childbirth, almost half of the women who took maternity leave are no longer in the labor market. It is noteworthy that, according to Law 14,020, women should have job stability from the date of pregnancy confirmation up to 5 months after childbirth. Data from the same study reported that almost half (45%) of the female workers return to work early, although the law allows them to take a leave of up to 4 months. Early return is justified by fear that they may lose their positions or, in some cases, even their jobs. According to the Federal Constitution, maternity leave guarantees 120 days of leave for mothers [8]. This period begins on the first working day after childbirth. It can be extended to 20 days, if the company is registered in the Citizen Company program [19, 21].

Our study has some strengths and limitations. The study was conducted in public maternity hospitals where the population of women had a lower level of schooling and family income, which could directly influence knowledge about their rights. Therefore, generalization of the findings can be limited. Similarly, as a cross-sectional study, it has limitations in establishing causal inference. The paucity of studies in the literature limited the comparison of our findings. Moreover, we excluded women with severe neonatal outcomes (fetal or neonatal death) due to ethical concerns. Conversely, the present study was conducted in maternity hospitals in different Brazilian regions with an adequate sample size that allowed an exploratory analysis of the subject. However, future studies with better representativeness are needed to assess the influence of the degree of knowledge and perception of violation on maternal and neonatal outcomes. During the interviews, study participants were offered a booklet on labor rights. This informative action can be adapted in hospital units and distributed to pregnant women during prenatal care or even in the puerperium. Moreover, it can also prepare health professionals to assist women in this process and formulate new educational policies aimed at women’s needs.

## Conclusion

A small proportion of women knew most of their employee rights, and a large proportion reported labor right violation, especially the most vulnerable population. Our findings suggest the need for improvements on policies to protect women’s rights, including educational measures, especially among the most socially vulnerable population. Moreover, surveillance to prevent violation to labor rights is required, particularly throughout pregnancy.

## Supplementary Information

Supplementary Material 1.

## Data Availability

The datasets generated and/or analysed during the current study are not publicly available due ethical and technical reasons but are available from the corresponding author on reasonable request. The study group is still conducting ancillary analyses of other topics related to this initiative, and that Ethical approval for the study did not consider the public availability of the information. Therefore, the data will only be made available upon request and under additional revision by the Ethical Review Board.
