# Supplementary material for: Knowledge and perception of labor rights violations among postpartum women in three Brazilian maternity hospitals: a cross-sectional study
Source: BMC Pregnancy Childbirth. 2025 Nov 14;25:1209. doi: 10.1186/s12884-025-08317-1 (PMC12619469; doi:10.1186/s12884-025-08317-1)
Supplement: Supplementary file 1 — Supplementary Material 1. [file 12884_2025_8317_MOESM1_ESM.docx]

**Supplementary Material**

Table S1: Labor Rights Related to Postpartum Pregnancy Cycle

| Labor right | Description | Knowledge Assessed | Violation Assessed |
| --- | --- | --- | --- |
| Article 5 | All work of equal value will correspond to equal salary, without sex discrimination. | X | X |
| Article 13 | The Work and Social Security Card is mandatory for the exercise of any employment, including rural, even on a temporary basis, and for the self-employed exercise of paid professional activity. | X | X |
| Article 58 | The normal working time for employees in any private activity shall not exceed 8 (eight) hours per day, provided that no other limit is expressly set. | X | X |
| Article 67 | Every employee is guaranteed a paid weekly rest of twenty-four consecutive hours, preferably on Sundays. | X | X |
| Article 129 | Every employee will be entitled to an annual vacation, without the loss of remuneration. | X | X |
| Article 482 | Just cause for dismissal or termination of the employment contract by the employer includes: act of misconduct; incontinence of conduct or bad procedure; habitual trading for personal reasons or unrelated without permission from the employer, and where it constitutes an act of competition to the company for which the employee works, or is detrimental to the service; criminal conviction of the employee, passed in court, if there was no suspension of sentence execution; performance of their respective functions; habitual drunkenness or in service; violation of company secrets; act of indiscipline or insubordination; job abandonment; harmful act of honor or good fame practiced in the service against any person, or physical offenses, under the same conditions, except in case of self-defense, of self or other; act ineffectual in honor or good fame or physical offenses committed against the employer and superiors, except in case of self-defense, of self or other; constant practice of gambling; loss of the qualification or requirements established by law for professional practice, due to the employee's intentional conduct. Sole paragraph - It is also a just cause for employee dismissal, duly proven in an administrative investigation, of acts attentive to national security. | X | X |
| Article 391 | It is not a good reason for termination of a woman's employment contract if she has contracted marriage or is in a state of pregnancy. | X | X |
| Article 392 | The employee is guaranteed, during pregnancy, without the loss of salary and other rights: | X | X |
| Article 392-1 | I - Transfer of function, as required by health conditions, ensuring the resumption of the previously performed function, immediately after returning to work;  II - Exemption from working hours for the time necessary to perform at least six medical consultations and other complementary exams. | X | X |
| Article 392-2 | The pregnant employee is entitled to maternity leave of 120 (one hundred and twenty) days, without the loss of employment or salary. | X | X* |
| Article 392-3 | The employee must, upon medical certificate, notify her employer of the date she started her leave from work, which may occur between the 28th (twenty-eighth) day before delivery and her return to work. | X | X* |
| Article 392-4 | Rest periods, before and after delivery, may be increased by 2 (two) weeks each, upon medical certificate. | X | X* |
| Article 395 | In case of non-criminal abortion, proven by official medical certificate, the woman will have a paid rest of 2 (two) weeks, assured of the right to return to the function she held before her removal. | X |  |
| Article 393 | During the period referred to in Article 392, the woman will be entitled to full pay and, when variable, the salary will be calculated according to the average of the last 6 (six) months of work, as well as the rights and advantages acquired, and she is also allowed to revert to the previously held function. | X | X* |
| Article 396 | To breastfeed the child, even in case of an adoptive mother, until the child is 6 (six) months old, the woman will be entitled, during the work day, to 2 (two) special half-hour rests each. | X | X* |
| Article 400 | Places that provide childcare for the children of employees during the breastfeeding period must have at least one nursery, a breastfeeding room, a diet eaten kitchen and a sanitary facility. | X | X* |

Labor right with (*) were considered only for multiparous women.

Source: Ministry of Labour and Employment - Consolidation of Labor Laws - CLT (2017) (7).
